# Vogt–Koyanagi–Harada Syndrome: Clinical Features, Immunogenetic Predisposition and PD-1 Inhibitor-Induced Forms—A Comprehensive Review

**DOI:** 10.3390/jcm15093490

**Published:** 2026-05-02

**Authors:** Sara Małgorzata Orłowska, Łukasz Bednarczyk, Kamal Morshed, Mateusz Tyniec, Paweł Olczyk

**Affiliations:** 1 Department of Health Sciences and Physical Culture, Student Scientific Association of Otolaryngology and Laryngological Oncology, Student Scientific Association “FarMedLab”, Radom University, Chrobrego 27, 26-600 Radom, Poland; bednarczyk.lukasz21@gmail.com; 2 Clinical Department of Otolaryngology and Laryngological Oncology, Mazovian Specialist Hospital in Radom, Radom University, 5 Aleksandrowicza Street, 26-617 Radom, Poland; k.morshed@urad.edu.pl (K.M.); m.tyniec@urad.edu.pl (M.T.); 3 Faculty of Medical Sciences and Health Sciences, Radom University, Chrobrego 27, 26-600 Radom, Poland; p.olczyk@urad.edu.pl

**Keywords:** Vogt-Koyanagi-Harada disease, uveitis, immune checkpoint inhibitors, immune-related adverse event, immunopathogenesis, genetic predisposition

## Abstract

Vogt–Koyanagi–Harada syndrome (VKH) is a rare granulomatous autoimmune disease characterised by a systemic immune response directed against melanocytes. This multisystem condition primarily affects organs that are rich in melanocytes, such as the eyes, inner ear, meninges and skin. VKH might be responsible for the development of chronic uveitis and permanent visual impairment, particularly in cases where a diagnosis is delayed and treatment is not administered in a timely manner. A key factor in its pathogenesis is the loss of immune tolerance to melanocytes, driven by a T-cell–mediated immune response and genetic susceptibility, including the presence of HLA-DRB1*04 antigens. In recent years, immune checkpoint inhibitors (ICIs) have become the standard treatment in oncology, including non-small cell lung cancer and unresectable melanoma. However, it should be noted that their utilisation carries with it the potential for immune-related adverse events, including rare cases of VKH-like uveitis. The objective of this review is to outline the clinical features of VKH syndrome, examine current diagnostic and treatment approaches, and emphasise the immunopathological mechanisms associated with drug-induced forms of VKH, with a particular focus on programmed cell death protein 1 (PD-1) inhibitors. The article also includes an analysis of the genetic, epigenetic, and environmental factors that predispose individuals to the disease. This analysis facilitates a deeper understanding of the pathogenesis of the disease and assists in the identification of patients at increased risk of drug-induced VKH manifestations.

## 1. Introduction

Vogt–Koyanagi–Harada syndrome (VKH) is a rare multisystem disorder that may occur as a side effect of immunotherapy used in the treatment of malignancies.

The condition is characterised by the presence of uveitis and exudative retinal detachment [1,2]. According to the definition of rare diseases, these conditions are often chronic and progressive, may be life-threatening, and affect a small percentage of the population compared to other diseases [3]. VKH syndrome is frequently associated with neurological, skin, and otolaryngological symptoms [1,2]. The fundamental principle underlying VKH pertains to the loss of immune tolerance to melanocytes, which are present in the cerebrospinal membranes, skin, inner ear, and eyes [4].

The incidence of VKH varies across different ethnic groups and is also dependent on genetic predisposition, as indicated by *HLA* markers [4,5]. VKH is a disease that primarily affects Asian populations (East and South Asia), but it also affects residents of Latin America and the Middle East [1,2,5,6,7]. In India, this syndrome is the most prevalent cause of uveitis [4]. According to statistical data, VKH is the leading cause of uveitis in India, manifesting at a rate of 21.08% [6]. In the United States, VKH syndrome accounts for approximately 4% of uveitis referrals, while in Japan it accounts for 8% [6]. According to the literature, the annual incidence of VKH in the United States is reported to range from 1.5 to 6 new cases per million people [5,8]. The observed variations in the incidence of VKH are believed to be attributable to genetic factors. This hypothesis is supported by studies of identical twins who report onset of the disease at the same time [4].

Epidemiological data from a retrospective study indicate that VKH is more prevalent in populations with darker skin pigmentation, including Asians, Latinos, African Americans, and people of Indian descent, while it remains relatively rare in European populations [9]. Studies suggest that women are more likely than men to develop the disease (at a ratio of 3:2 or 2:1) [2,4,6]. The typical age of onset is between the second and fifth decades of life [6]. However, in Asian populations, the peak incidence occurs earlier, around the age of 30 [7].

The clinical course of the disease varies and depends on both the ethnic origin and age of the patients. In people of Asian descent, VKH syndrome often has a more aggressive course. In contrast, in Caucasians, the disease generally follows a milder course, with a lower risk of serious complications, including permanent visual impairment [8,10]. In children, VKH tends to be severe and chronic, often leading to subretinal fibrosis [4]. In older patients over 65 years of age, however, optic disc hyperemia and choroidal detachment are more common [4].

Recently, there have been an increasing number of reports of VKH syndrome or uveitis with a VKH phenotype caused by immune checkpoint inhibitors (ICIs). This is especially common following the use of PD-1 receptor inhibitors, such as pembrolizumab or nivolumab [2,11]. In cases of drug-induced VKH, symptoms often develop rapidly, even during ongoing cancer treatment, creating a significant diagnostic and therapeutic challenge for clinicians. Therefore, it is important to further explore the role of the programmed cell death protein 1 (PD-1)/programmed death ligand 1 (PD-L1) immune checkpoint axis in maintaining immune tolerance to melanocytes and identify factors that predispose individuals to this rare and potentially severe complication [2,11,12].

This article aims to provide a comprehensive review of the current knowledge on Vogt–Koyanagi–Harada syndrome, covering both the classic clinical features of the disease and an analysis of published cases of uveitis with a VKH phenotype caused by PD-1 receptor inhibitors. This study primarily aims to identify risk factors and conditions that predispose individuals to developing drug-induced VKH, with a particular focus on immunogenetic mechanisms linked to IL-23/IL-17 pathway polymorphisms, *HLA* gene variation, and other genes and epigenetic factors involved in immune response regulation. Examining these factors seeks to enhance understanding of the pathogenesis of VKH and to provide a basis for identifying patients at increased risk for this complication during treatment with ICIs.

## 2. Materials and Methods

This article is conceived as a narrative review aimed at summarizing the current knowledge regarding VKH, with particular emphasis on its clinical presentation, immunogenetic background, and forms induced by ICI therapy. The primary objective of this review was to integrate contemporary evidence concerning the pathogenesis, diagnosis and treatment of VKH, as well as to highlight the mechanisms underlying drug-induced VKH-like uveitis associated with PD-1 inhibitors.

To ensure a comprehensive and evidence-based synthesis of the available literature, a structured search strategy was applied. A comprehensive search of relevant articles was conducted using major electronic databases, including PubMed/MEDLINE, Scopus, and Google Scholar. The search encompassed publications from January 2000 to January 2026, thereby incorporating both historical descriptions of the disease and the most recent studies concerning immunotherapy-related VKH manifestations. The search strategy employed combinations of keywords and Medical Subject Headings (MeSH), including “Vogt–Koyanagi–Harada disease”, “VKH syndrome”, “uveitis”, “immune checkpoint inhibitors”, “PD-1 inhibitors” and “immunogenetics”.

The selection of literature focused primarily on peer-reviewed publications written in English, including review articles, clinical studies, cohort analyses, case series and case reports that provided relevant insights into VKH pathophysiology, diagnostic approaches, and treatment strategies. A particular emphasis was placed on publications that delineated immune-related adverse events associated with ICIs, particularly PD-1 inhibitors such as pembrolizumab and nivolumab. The articles were subjected to a rigorous evaluation process that entailed the assessment of their scientific relevance and their contribution to the advancement of knowledge concerning the clinical and immunological facets of VKH. As this work constitutes a narrative synthesis of previously published data, no new clinical data were collected or analyzed. Consequently, ethical approval and informed consent were not required for this study, as the review was based solely on previously published literature.

## 3. Pathogenetic Determinants and Risk Factors for Vogt–Koyanagi–Harada Syndrome

The cause of VKH is not fully understood. As indicated by histopathological and immunohistochemical studies, T lymphocytes, especially T helper 17 (Th17) cells, which are the primary initiators of the autoimmune disease process, may play a significant role in the development of this condition [13,14]. It was found that VKH results from a T-cell response directed against melanocyte antigens in the choroid, meninges and cochlea [14]. Autoreactive T lymphocytes targeting tyrosinase and other melanocyte-specific proteins, including CD4+ cells infiltrating ocular tissues, have been identified in affected patients, although most evidence derives from experimental and observational studies [13,14]. The confirmed targets of this response in the pathogenesis of VKH are proteins involved in melanocyte differentiation [13].

There are two key elements in the initial phase of the disease, i.e., genetic predisposition and environmental factors. These factors are believed to influence susceptibility to disease development and timing of onset, although the exact causal relationships remain incompletely defined [13,15]. Genetic and environmental factors determine susceptibility to the development of the disease and the timing of its occurrence initiation. In the next stage of the disease, antigen-presenting cells (APCs) mature and display antigenic structures to naive T lymphocytes. In VKH syndrome, a marked shift in the immune response toward Th1 and Th2 lymphocytes has been observed. Cytokines, chemokines, and other mediators secreted by Th1, Th17 and other immunocompetent cells create a specific immune environment that may facilitate activation of effector lymphocytes, leading to an attack on melanocyte-rich tissues [15].

### 3.1. Genetic Predisposing Factors

#### 3.1.1. Polymorphisms of Inflammatory Response Regulatory Genes

Interleukins have been proposed as potential contributors to genetic susceptibility in VKH, as they play key roles in regulating immune responses [16]. Associations between VKH and polymorphisms in genes involved in interleukin-12 (IL-12) and interleukin-17 (IL-17) pathways have been reported in several studies [1,4,17]. However, most of these studies are based on limited cohorts, narrative reviews and population-specific analyses, which limits generalizability.

**Figure 1:**
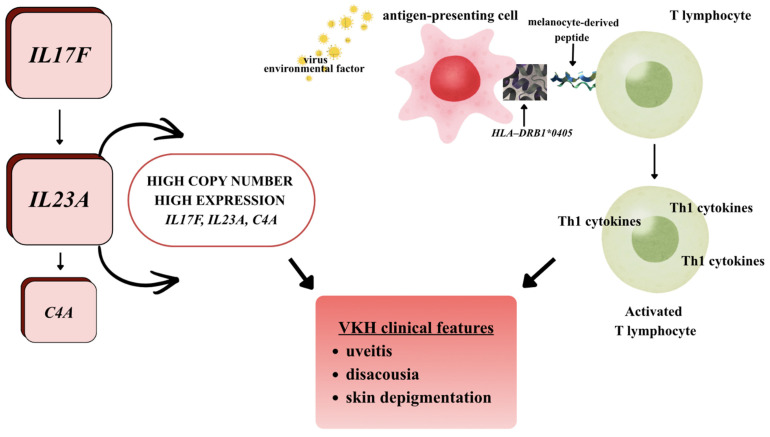
The role of IL-23/IL-17 and HLA-DRB1*0405 in the pathogenesis of Vogt–Koyanagi–Harada syndrome.

The IL-12/Th1 and IL-23/IL-17 axes have been suggested to be involved in disease pathogenesis (Figure 1) [16,17].

Interleukin 12 may contribute to differentiation of naive T lymphocytes into Th1 lymphocytes, and the *Crs3212227* allele of the *IL-12B* gene is a potential risk factor for developing VKH disease. In turn, increased expression of interleukin 17 has been associated with intraocular inflammation in patients with VKH and Behçet’s disease. Additionally, it has also been demonstrated that the *rs763780* polymorphism of the *IL-17F* gene correlates with the occurrence of VKH in the Chinese Han population, where the TT genotype raises susceptibility to the disease and the C allele has protective functions [16].

Another important cytokine identified in the context of the development of autoimmune diseases is interleukin 23, which promotes an increase in IL-17 production by CD4+ lymphocytes, thereby potentially contributing to chronic inflammation. It has been shown that elevated IL-23 levels were observed in the serum of patients with VKH and active uveitis [16].

Interleukin 27 is expressed in photoreceptors and retinal ganglion cells. This cytokine encourages the differentiation of naive T lymphocytes towards Th1, while inhibiting their differentiation into Th17, which results in mutual antagonism between Th1 and Th17 populations—both of which are involved in the pathogenesis of uveitis [16].

#### 3.1.2. Copy Number Polymorphism and Other Immune System Genes

Initial research on the pathogenesis of VKH focused primarily on human leukocyte antigen (*HLA*) polymorphisms located on the short arm of chromosome 6 (6p21.3). VKH shows a strong correlation with *HLA-DR4* alleles and *DRB1/DQA1* haplotypes. However, these findings are largely based on case–control studies in specific populations [18]. Genetic analyses have revealed that, in addition to single nucleotide polymorphisms (SNPs), which involve single nucleotide changes (accounting for less than 1% of the genome), structural variants, such as copy number polymorphisms (CNVs), which cover 5–10% of the genome and may explain 10–20% of disease heritability, are also significant [18]. CNVs influence the expression levels of immune system effector proteins, leading to the deregulation of immune tolerance mechanisms and promoting the development of autoimmune diseases, including VKH. Studies of CNVs in VKH syndrome have identified notable alterations in the *IL17F*, *IL23A*, and *C4A* genes, but these findings are preliminary and require validation in larger, multi-ethnic cohorts (Figure 1) [19].

The figure illustrates the potential multifactorial pathogenic mechanism of VKH, which considers the interaction between genetic predisposition and environmental factors that lead to the activation of autoreactive T lymphocytes targeting melanocyte antigens. Genetically, an increased copy number and higher expression of the *IL17F*, *IL23A*, and *C4A* genes are evident, with these genes playing key roles in regulating the inflammatory response. IL-23 (encoded by *IL23A*) has been shown to support the maintenance and proliferation of Th17 lymphocytes, while *IL-17F* acts as an effector cytokine that amplifies inflammation. Enhanced activity of the IL-23/IL-17 pathway escalates autoimmune processes, recognised as a significant element in the pathogenesis of VKH. At the same time, activation of the complement system (*C4A*) may worsen the inflammatory response. An environmental factor, including a potential viral infection, triggers the maturation of APCs. In the presence of the predisposing HLA-DRB1*0405 allele, melanocyte-derived peptides are presented to CD4+ T cells. This mechanism may involve molecular mimicry, where sequence similarity between viral antigens and melanocyte proteins causes cross-reactivity and loss of immune tolerance. Activated T lymphocytes mainly differentiate towards Th1 and Th17 responses, secreting proinflammatory cytokines that initiate and sustain inflammation in melanocyte-rich tissues—the choroid of the eye, meninges, inner ear and skin. This immune response results in the characteristic clinical symptoms of VKH, including uveitis, hearing impairment (dysacusia), and skin discolouration resulting from the destruction of melanocytes [2,18,20].

Additional genetic associations have been reported in genes such as *CTLA4* and in GWAS-identified loci including *IL23R/C1orf141*, *EGR2/ZNF365/ADO*, and *HLA-DRB1/DQA1*. While these findings suggest polygenic susceptibility, most studies are limited by population specificity, and lack of replication [17,18,21].

The influence of SNPs in the etiopathogenesis of VKH SNP has been established. An example is the *CTLA4* gene, located on chromosome 2q33 in the *CD28* gene family. In the Chinese Han population, specific *CTLA4* polymorphisms have been found to be associated with the occurrence of VKH syndrome [17,18].

Genome-wide association studies (GWASs) have identified several non-*HLA* genes linked to VKH susceptibility, i.e., *IL23R/C1orf141* in the Singaporean population and *EGR2/ZNF365/ADO* in the Thai population [17,18,21]. In a GWAS study analysing over 2.2 million SNPs, three loci significantly associated with VKH susceptibility were identified: *IL23R–C1orf141, ADO–ZNF365–EGR2,* and *HLA-DRB1/DQA1* [21]. All these non-*HLA* genes are expressed in eye tissues, including the iris, ciliary body and choroid, underlining their potential role in the pathophysiology of uveitis [21].

Loss of immune tolerance to melanocytes results in the development of non-suppurative, granulomatous inflammation in the eye, inner ear, and skin [4]. In the eye, inflammation manifests as Dalen-Fuchs nodules beneath the RPE [4]. Histopathological analysis of Dalen-Fuchs nodules shows granulomas composed of histiocytes, hematoxylin and eosin staining reveals B and T lymphocytes in the choroid, confirming the loss of melanocytes [4].

### 3.2. Epigenetic Factors

#### 3.2.1. Variability of DNA Methylation Patterns and Interaction with the BTNL2, NOTCH4, RIBC2, TNXB, and AGPAT2 Genes

In VKH syndrome, DNA methylation turns out to be most strongly associated with the *HLA* region. These findings should be interpreted as associations rather than confirmed pathogenic mechanisms, as most studies are exploratory and based on limited cohorts. Twelve CpG regions (specific DNA sequences susceptible to epigenetic changes that influence gene activity) with abnormal methylation were confirmed (three located in the *HLA* region and nine in non-*HLA* gene regions). Three associated CpG sites were identified in the *HLA* region: *cg04026937* and *cg18052547* in *HLA-DRB1* and *cg13778567* in *HLA-DQA1* [22].

Beyond *HLA* genes, research has also identified methylation variability in other immune and inflammation-related genes. Five CpG sites with significantly altered methylation were located in the *BTNL2* gene, which lies at the border between *HLA* class II and III regions. *BTNL2* shares homology with the B7 family and plays a role in T-cell co-signaling. Polymorphisms in *BTNL2* are linked to inflammatory conditions such as psoriasis. In VKH, increased expression of *BTNL2* mRNA has been observed in whole peripheral blood, which may suggest a negative feedback mechanism during the disease course.

Another gene in which the cg22155039 hypomethylation site has been identified is *NOTCH4*. An activation of the *NOTCH4* pathway enhances the inflammatory response and leads to increased mRNA expression in VKH patients [23,24].

The *AGPAT2* gene has been confirmed as a factor that predisposes to sarcoidosis, and current research indicates its potential influence on the development of VKH. It is located on chromosome 9 and is involved in converting lysophosphatidic acid to phosphatidic acid during the de novo synthesis of glycerolipids [23,24].

Other genes still needing extensive research to understand their role in VKH include *RIBC2* and *TNXB*. It has been shown that CpG sites in these genes also exhibit variable methylation, suggesting a potential effect on immune response and inflammatory processes in the disease [23,24].

Abnormal DNA methylation patterns in *HLA* and non-*HLA* genes may act as biomarkers of active VKH and influence its development by modulating the expression of genes associated with the immune response. However, their functional role in VKH remains largely speculative and requires further investigation [22,23,24].

#### 3.2.2. MicroRNA (miRNA)

MicroRNAs are short, non-coding RNA molecules that act as post-transcriptional regulators of gene expression by binding to the 3′UTR region of target mRNA, leading to its degradation or inhibition of translation. They form an additional layer of epigenetic regulation of gene expression. Patients with VKH have been shown to exhibit altered expression of specific miRNAs that influence signaling pathways linked to Th1 and Th17 lymphocyte activation and proinflammatory cytokine production, although the precise functional and causal relationships in human VKH remain to be fully established [18,23].

In VKH, increased expression of GATA3, IL-4, and TGF-beta is observed, indicating a role for DNA methylation in regulating the immune response, which may reflect immune dysregulation, although the upstream epigenetic drivers, including DNA methylation changes, remain incompletely understood. Moreover, CNVs involving miR-23a, miR-146a, and miR-301a have been associated with susceptibility to VKH disease [18]. Specifically, miR-23a overexpression in ARPE-19 cells activates pro-inflammatory cytokine IL-6, which contributes to the persistence of immune disorders and chronic inflammation [18,23].

The significance of miRNAs in the pathogenesis of uveitis is also supported by studies conducted using an experimental autoimmune uveitis model, which demonstrated dynamic changes in miRNA expression correlating with IL-17 levels and Th17 cell activity. These findings suggest that miRNAs are vital in modulating the immune response in both VKH and experimental uveitis models. However, these associations are based on limited datasets and require validation in larger cohorts for substantiation [23].

### 3.3. Interaction Between Environmental Factors and Microbiot. The Phenomenon of Molecular Mimicry in VKH

VKH is triggered by environmental factors in genetically predisposed individuals. Factors such as infections, seasonality, and vitamin D levels have been proposed, but supporting evidence is limited and not consistent across studies [25]. Increasingly, the patient’s gut microbiome is being identified as an important risk factor that influences the course of uveitis. Mechanistic hypotheses propose that microbial metabolites may influence immune regulation and epigenetic modifications, but these pathways remain largely theoretical in the context of VKH [25,26]. Infectious and non-infectious uveitis are associated with dysregulation of numerous genes and microRNAs. The expression of these genes requires chromatin remodelling, which is modulated by both commensal and pathogenic bacteria [26]. It has been demonstrated that short-chain fatty acids (SCFAs)—lactate, acetate, propionate—can affect the host’s chromatin. For this reason, it is suggested that the gut microbiota is an important regulator of epigenetic processes and plays a significant role in the pathogenesis of uveitis [25,26]. It turns out that the intraocular environment is not sterile. Using quantitative PCR, transmission electron microscopy (negative staining), direct cultures, and high-throughput sequencing methods, the presence of intraocular microbiota has been demonstrated. When the intestinal barrier is damaged or the blood-retina barrier is disrupted, this leads to the penetration of bacteria, and their metabolites can penetrate into systemic circulation and into the eye, leading to secondary chromatin modifications [22,25].

In addition, various intestinal microorganisms can directly influence the innate immune maturation system by modulating Toll-like receptors (TLRs) and other immune receptors, playing a key role in the pathogenesis of many autoimmune diseases [23]. TLRs are capable of recognising ligands of pathogenic microorganisms. In VKH, there is an increased expression of TLR-3 and TLR-4 receptors. A significantly higher proportion of *Ramularia*, *Alternaria,* and *Rhizophagus* was found at the genus level in patients with active VKH [23].

In patients diagnosed with VKH, the predominance of Gram-negative bacteria has been associated with increased activity of lipopolysaccharide (LPS) biosynthesis pathways. In contrast, LPS binding to TLR4 receptors can induce a strong inflammatory response, leading to excessive activation of the LPS/TLR4 axis in patients with active VKH [23]. In addition, patients with VKH have been observed to have increased oxidative phosphorylation pathways, and methane production by *Bifidobacterium* or *Clostridium* was significantly reduced. According to studies in animal models, methane plays an important role in regulating inflammatory processes and protecting against oxidative stress [23].

In patients with VKH, environmental factors and genetic predisposition interact with intestinal microbiota disorders, leading to the modulation of TLRs, epigenetic changes, and increased inflammation. In addition, changes in the intestinal microbiome in patients with VKH correlated with genotypes associated with disease susceptibility [22].

The immune cascade described in earlier sections of this article may be triggered by environmental factors, such as viral infections or skin injury. Evidence for molecular mimicry between viral antigens (cytomegalovirus, Epstein–Barr virus, SARS-CoV-2) and melanocyte-associated proteins has been reported in isolated studies and case reports [13,14]. While these mechanisms are biologically plausible, the supporting evidence is limited and heterogeneous, and their role in disease initiation remains unproven in large clinical cohorts [1,4,13].

Cytomegalovirus, which stimulates T cells to interact with tyrosinase, has been shown to initiate VKH [14]. The authors [13] pointed out that fragments of two different proteins—tyrosinase (position 462) and the cytomegalovirus envelope glycoprotein, share an identical sequence of six key amino acids that are crucial for the binding of the HLA-DRB1*0405 antigen. T lymphocytes involved in the recognition of viral proteins can be reactivated by a fragment of human tyrosinase, which causes a cross-reaction and induces the autoimmune process responsible for VKH in genetically predisposed individuals [13]. The importance of HLA DRB1*0405 in susceptibility to VKH is confirmed by a study in which peripheral blood lymphocytes from HLA-DRB1*0405-positive patients recognised a much broader repertoire of peptides derived from melanocytes than cells from patients without genetic predisposition [13].

The literature reports cases of VKH patients in whom Epstein–Barr virus was isolated from the vitreous body and a correlation between the occurrence of VKH and SARS-CoV-2 infection was found [1,4,14]. It was pointed out that molecular mimicry between viral antigens and melanocyte-related structures may promote the induction of an immune response leading to the formation of antibodies responsible for the development of symptoms characteristic of VKH [14].

## 4. Vogt–Koyanagi–Harada Syndrome—Clinical Picture, Diagnosis and Current Treatment Options

### 4.1. Clinical Picture

The initial ophthalmic symptoms of VKH include redness, ocular pain, decreased visual acuity, and photopsia. These symptoms are often accompanied by headaches, dizziness, nausea, tinnitus, and hearing disorders. Vestibular-auditory symptoms are present in 75% of patients, and sensory hearing loss primarily affects high frequencies [14].

An ophthalmological examination reveals inflammation of all parts of the uvea or swelling of the optic disc. The presence of corneal deposits, Busacca and Koeppe nodules is characteristic. In the vast majority of patients, the symptoms also involve the contralateral eye within two weeks [14]. There are four stages of VKH, which are described in Table 1.

### 4.2. Diagnosis

The diagnosis of VKH is based on the clinical picture, confirmed by laboratory and imaging findings [4]. The physical examination should focus on the number of prior disease episodes, the presence of eye pain, hearing disorders or neurological symptoms [4]. From a practical perspective, diagnostic evaluation should distinguish between modalities essential for initial diagnosis and those most useful for monitoring disease activity and progression [4,27].

Fluorescein angiography and indocyanine green angiography (ICGA) remain key tests for both diagnosis and monitoring, as they allow assessment of choroidal inflammation and treatment response [4]. These techniques are particularly valuable in the acute phase, when inflammatory activity is most pronounced, but they also play an important role in detecting subclinical disease during follow-up [4].

However, in routine clinical practice, optical coherence tomography (OCT) has become the most accessible and widely used first-line tool, as it is a non-invasive imaging technique that allows in vivo evaluation of the retina and choroid [1]. In the acute phase, OCT typically reveals serous retinal detachments and increased choroidal thickness, reflecting active inflammation [4,27]. In patients in the recurrent or chronic stage, OCT often reveals a reduction in choroidal thickness and volume, reflecting the prolonged inflammatory process [4,27].

OCT angiography (OCTA) is becoming increasingly important for monitoring VKH, as it enables detailed assessment of choriocapillaris perfusion and other microvascular changes, making OCTA a useful tool for longitudinal follow-up rather than primary diagnosis [27]. Recent studies have shown that in patients in the acute stage of the disease, treated with corticosteroids with or without immunosuppressive therapy, OCTA does not reveal any changes in the retinal capillary plexus, while in most patients, diffuse areas of reduced flow were observed in the choroidal capillary layer and in the Sattler layer [27]. These changes, confirmed by other imaging studies such as OCT and ICGA, are interpreted as areas of no flow [28]. It has been shown that OCTA in patients with acute VKH can be used to monitor treatment response. OCTA performed after treatment initiation suggested a reduction in the number of multiple areas of no flow in the choroidal capillaries [28].

Overall, current evidence suggests that fluorescein angiography, ICGA, OCT, and OCTA are the principal imaging modalities in VKH, while other techniques, including EDI-OCT, SS-OCT, and FAF, may provide additional, stage-dependent insights [4,27,28,29].

Enhanced depth imaging OCT (EDI-OCT) provides additional structural information and is particularly valuable for quantitative monitoring of disease activity [29]. This technique is a non-invasive, repeatable, and intuitive imaging technique that allows precise in vivo measurement of subfoveal choroidal thickness (SFCT) and central macular thickness (CMT). This method provides significant clinical value for the assessment of structural changes in the choroid and macula in patients with VKH [29]. Using EDI-OCT, clinicians can detect disease-related alterations in choroidal thickness depending on the stage of VKH. In the early stages of the disease, EDI-OCT typically reveals increased choroidal thickness, which gradually decreases during effective immunosuppressive therapy. In contrast, in late or recurrent stages, or in cases of inadequate treatment, prolonged inflammation may lead to choroidal thinning and atrophy. Furthermore, EDI-OCT has been shown to reveal damage to the choroidal-retinal layer (choriocapillaris), which is more pronounced in chronic VKH and correlates with persistent visual impairment [30].

The next diagnostic method in the imaging evaluation of VKH syndrome is swept-source OCT (SS-OCT), which is particularly useful for assessing the acute phase of the disease [31,32]. SS-OCT facilitates enhanced visualisation of the choroid and choriocapillaris layers over a broader field of view, enabling both monitoring of treatment response and prediction of VKH recurrence [33,34]. The thickening of the choroid and irregularities of the retinal pigment epithelium (RPE) observed on SS-OCT may indicate a more severe course of the disease and necessitate modification of therapy [31,32,33]. This technique is performed using a system operating at a central wavelength of 1050 nm, providing an axial resolution of 2.6 µm and a transverse resolution of 14 µm. The utilisation of near-infrared light reduces scattering and allows for better visualisation of deep ocular structures, including the posterior vitreous cortex and choroid [31,32]. In contrast to spectral-domain OCT, the utilisation of swept-source technology obviates the necessity for a spectrometer, eliminating the limitations associated with a nonlinear sensitivity profile and resulting in superior image quality in both the vitreous and choroidal layers [31,32,33].

Another imaging method used to assess VKH is fundus autofluorescence (FAF). This test is reflective of functional changes in the RPE associated with lipofuscin accumulation, allowing the identification of RPE damage not visible in routine ophthalmoscopic examination, as commonly observed in patients with VKH. An additional advantage of FAF is the ability to determine the extent of posterior segment involvement, especially when using wide-field techniques [34,35]. In the acute phase of VKH, the FAF image is most often characterised by generalised autofluorescence enhancement due to transient RPE dysfunction [36]. In certain instances, areas of diminished autofluorescence signal have been observed, which appear to be associated with the presence of exudative retinal detachments in the early stages of the disease. This is likely attributable to the obstruction of the RPE signal by subretinal fluid [34]. However, these changes do not occur in all areas of detachment and usually regress gradually after intensive corticosteroid therapy [35].

**Figure 2:**
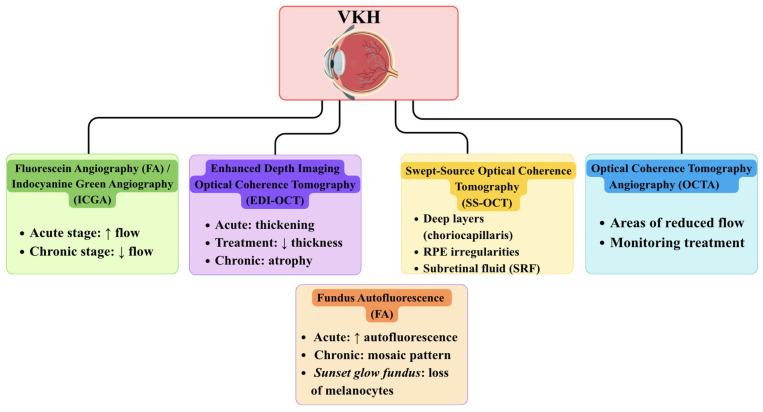
Multimodal ocular imaging in Vogt–Koyanagi–Harada disease [1,4,31,32,33,34,35,36]. ↑—increase in the parameter value; ↓—decrease in the parameter value.

A divergent FAF pattern has been observed in patients presenting in the later stages of VKH. In this group of patients, an irregular, mosaic pattern of areas of increased and decreased autofluorescence is more common, especially in locations previously affected by subretinal fluid. Areas of hypoautofluorescence may correspond to permanent RPE damage, such as peripapillary atrophy or atrophic and pigmented scars, while foci of hyperautofluorescence have been described in the course of cystoid macular edema. The characteristic sunset glow fundus image, which is characteristic of chronic VKH, is not associated with significant changes in the autofluorescence signal. This finding suggests that choroidal melanocyte loss plays a more dominant role than RPE damage (Figure 2) [34,36].

Laboratory investigations play a supportive role. In the prodromal phase of VKH, cerebrospinal fluid examination reveals pleocytosis, which may persist for several weeks after symptoms have subsided [4]. It was found that elevated serum IgE levels in patients in the acute phase of VKH correlated with increased vascular permeability and exacerbation of the inflammatory process [4].

### 4.3. Treatment

Management of VKH is highly dependent on early therapeutic intervention, with treatment strategies guided by disease stage. Systemic corticosteroids remain the first-line therapy, typically initiated as high-dose intravenous pulses in the acute phase, which has been shown to reduce the risk of severe vision loss [4,11,14]. Once the patient’s condition stabilises in the acute phase of the disease, gradual tapering of glucocorticosteroids is recommended [11].

A key concept in VKH management is the “therapeutic window of opportunity” [4,27]. According to this concept, the speed of diagnosis and early initiation of therapy—preferably in the acute stage of the disease before the anterior segment of the eye is affected—are of utmost importance for effective treatment of the patient. In this way, the chronic and recurrent phases of the disease can be prevented and the risk of complications reduced [28]. Observational cohort data suggest that a delay in starting treatment by 3–4 weeks from the onset of symptoms is associated with a higher risk of recurrence and complications, regardless of the steroid treatment implemented [28].

Increasing evidence supports the early introduction of combined therapy, including systemic corticosteroids and immunomodulatory agents, particularly in patients at higher risk of severe disease. This approach has been associated with a lower incidence of chronic changes such as “sunset glow fundus” and improved long-term outcomes. However, these findings are based on limited evidence, largely derived from observational studies, and should be interpreted with caution. Further large-scale, prospective studies are needed to better establish their clinical relevance and long-term efficacy [28].

In cases of insufficient response or corticosteroid dependence, second-line therapies include calcineurin inhibitors such as cyclosporine, anti-TNF drugs, and mycophenolate mofetil [1]. A growing body of studies is evaluating the effect of biological treatment in patients with chronic VKH, especially in cases where other treatments have been ineffective [28]. Adalimumab treatment has shown promising results, with reported reductions in inflammatory activity on indocyanine green angiography and a reduction in choroidal thickness in patients with VKH, although these observations are largely based on small retrospective studies. The effectiveness of adalimumab treatment has also been observed in patients with sunset glow fundus lesions [28]. A Japanese retrospective study evaluated the effect of adalimumab treatment in patients with chronic VKH, showing significant reductions in subretinal choroidal thickness and a decrease in the average daily dose of corticosteroids after six months of adalimumab therapy [28]. The current evidence supporting the use of adalimumab in refractory or chronic VKH remains moderate and is primarily based on case reports and small case series rather than randomized controlled trials [1,28].

Local therapies, including periocular or intravitreal corticosteroid administration, may be used as adjunctive treatment for ocular inflammation but do not address systemic manifestations of the disease [4,28]. Subconjunctival triamcinolone has been reported to be effective in some patients and led to stabilisation of VKH symptoms without recurrence in 78% of patients [4]. However, this method is associated with numerous side effects characteristic of glucocorticosteroids, such as cataracts and increased intraocular pressure [28]. Topical treatment is recommended mainly for patients receiving systemic treatment, as subtenon steroid administration is primarily effective for ocular symptoms and does not affect auditory or dermatological symptoms.

Management of complications, such as glaucoma, cataract, or subretinal fibrosis, may require surgical intervention [4].

The prognosis for VKH disease is generally good when appropriate treatment is initiated promptly. However, prognostic data are primarily derived from single-center retrospective cohorts, small case series, and narrative reviews, rather than randomized controlled studies. In addition, heterogeneity in diagnostic criteria and the inclusion of both acute and chronic cases in earlier studies further limits the precision and generalizability of prognostic estimates [1,4,11,28].

Studies indicate that early initiation of immunosuppressive therapy, preferably within the first two weeks of symptom onset, significantly improves visual outcomes and reduces the risk of chronic complications [11,28]. It is well-documented that, with the administration of timely and adequate therapy, 60–70% of patients experience substantial improvement in visual acuity. Delayed treatment, inadequate dosing or interruptions in therapy, on the other hand, are associated with persistent ocular inflammation, progressive choroidal atrophy, and a higher likelihood of permanent vision loss [11,28]. According to Zhang et al. [11], careful monitoring and rapid adjustment of immunosuppressive regimens in patients receiving ICIs can prevent irreversible ocular damage and further improve prognosis. Tugal-Tutkun et al. [28] emphasise that combination therapy with corticosteroids and additional immunomodulatory agents is often necessary in severe or recurrent cases to achieve optimal visual recovery.

Importantly, much of the available evidence—particularly in the context of checkpoint inhibitor-associated VKH—is based on case reports and small observational studies. This limits the strength of conclusions regarding treatment efficacy and long-term outcomes, and highlights the need for larger, prospective studies [4,11,28]

## 5. Vogt–Koyanagi–Harada Syndrome as an Immune-Related Adverse Event of PD-1 Inhibitor Therapy: Mechanisms and Pleiotropic Effects

Cancer cells can be recognised and eliminated by the immune system. However, the PD-1/PD-L1 pathway controls the development and maintenance of immune tolerance in the tumour microenvironment. PD-1 and its ligands, PD-L1 and PD-L2, regulate the activation, proliferation, and release of cytotoxic substances by T lymphocytes in the course of cancer [37]. Both PD-1 and PD-L1/L-2 inhibit the function of immune cells, limiting the body’s ability to mount an effective anti-tumour response and potentially allowing tumours to evade immune surveillance in tissues [38]. This mechanism is of particular relevance to Vogt-Koyanagi-Harada disease. In VKH, impaired function of the PD-1/PD-L1 axis-whether due to reduced PD-1 expression on T cells or decreased PD-L1 levels-leads to defective immune tolerance, resulting in uncontrolled T-cell activation and persistent inflammation in melanocyte-rich tissues such as the uvea, meninges, skin, and inner ear [38].

PD-1 is an immunosuppressive receptor with a molecular weight of approximately 50–55 kDa. It is found on the surface of B lymphocytes, T lymphocytes, natural killer cells, and myeloid cells [37]. Known as CD279, it belongs to the immunoglobulin superfamily and is a type I transmembrane glycoprotein [38].

The main role of PD-1 is to regulate T cell activity in tissues and inhibit their ability to induce cell death in cancerous conditions. In turn, the binding of PD-1 to PD-L1 inhibits T cell receptor (TCR) signalling and activity. Inhibition of T cell proliferation reduces cytokine production by cytotoxic CD8+ lymphocytes and a decrease in cytotoxic activity [39]. Overexpression of PD-L1 in cancer cells is associated with a poor prognosis in many types of cancer [37]. Activation of PD-1 shifts the metabolism of CD8+ lymphocytes from glycolysis to β-oxidation of fatty acids. This process leads to the production of reactive oxygen species, mitochondrial damage, and cell death [39]. The described PD-1-dependent suppression of T-cell proliferation and cytotoxicity is essential for maintaining peripheral tolerance. In VKH syndrome, disruption of this pathway—resulting from diminished PD-1 expression or function—allows autoreactive CD8+ lymphocytes to evade metabolic shutdown and persist within melanocyte-containing tissues, perpetuating the autoimmune inflammation that defines the condition [37,38,39].

**Figure 3:**
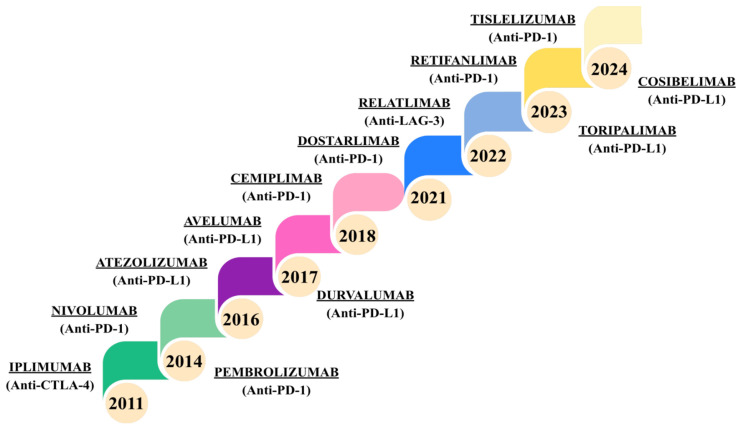
Timeline of FDA approvals for immune checkpoint inhibitors (2011–2024) [41].

PD-1/PD-L1 inhibitors are a class of anticancer drugs that block the function of checkpoint proteins on cell membranes. By inhibiting the PD-1/PD-L1 interaction, they enable the immune system to eliminate cancer cells [30,40]. PD-1 inhibitors have been shown to be highly effective in the treatment of metastatic melanoma. More than 50% of patients surviving ≥ 4 years achieve a durable response [39]. Between 2011 and 2024, the FDA approved thirteen ICIs (Figure 3) [41].

As shown by Raskov et al. [39], in some patients with cancer, a complete response to anti-PD-1 treatment can be achieved within 80 days in some cancer patients. However, not all patients achieve lasting therapeutic effects. According to Raskov et al. [39], anti-PD-1 therapy of malignancies is associated with immune-related adverse events that can affect multiple organ systems, including the digestive and endocrine systems. In rare cases, this immune activation can lead to autoimmune complications such as VKH-like syndrome. To reduce toxicity and increase the effectiveness of immunotherapy, polytherapy is being explored.

Anti-cancer immunotherapy has numerous pleiotropic effects, influencing various mechanisms of the immune system and the tumour microenvironment (TME) [42,43,44,45]. The tumour microenvironment is a complex ecosystem that plays a key role in the tumour evasion of the immune response [44].

One key aspect is the modulation of regulatory T cells (Tregs), which play an important role in maintaining immune system homeostasis by suppressing excessive immune responses [44]. In tumours, Tregs often accumulate in the tumor microenvironment, weakening the action of cytotoxic T lymphocytes (CTLs) and NK cells and promoting immunosuppression, which helps the tumour escape immune surveillance and, as a result, leads to metastasis [43,44]. Increased expression of the PD-1 ligand, PD-L1, is associated with increased infiltration of Tregs, myeloid-derived suppressor cells (MDSCs), and anti-inflammatory M2 macrophages, all of which have immunosuppressive properties [43]. Treg-rich tumours create an immunosuppressive environment that impairs the activity of immune cells and facilitates the development of metastases [44]. Studies confirm [43] that pembrolizumab, as an anti-PD-1 agent, strategically blocks the PD-1/PD-L1 interaction and prevents cancer cells from escaping.

Another element is the interaction with the TME, which consists of cancer cells, blood vessels, fibroblasts, macrophages, and immune cells. The cooperation between cancer cells and the surrounding stromal environment is crucial in modulating the growth and differentiation of cancer cells [42]. In the elimination phase, the immune system removes cancer cells and plays a protective role. In the equilibrium phase, tumour growth is chronic. During this stage, the degree of inflammation increases. In the final phase, cancer cells evade immune system detection. The use of ICIs, such as anti-PD-1 and anti-CTLA-4 antibodies, transforms the tumor microenvironment by enhancing T cell activity, reducing immunosuppressive signals, and promoting cytotoxic CD8+ T lymphocyte infiltration. While these effects improve antitumor immunity, they can also disturb immune tolerance to self-antigens, including melanocyte-associated antigens, thereby contributing to the development of VKH [42].

Another potential mechanism that may be involved in VKH induction is the modulation of intercellular communication via exosome secretion. Checkpoint blockade can alter the composition of exosomes, which may carry immunosuppressive or stimulatory molecules, promoting an aberrant immune response against melanocytes [42]. Exosomes secreted by mesenchymal stem cells in breast cancer contain TGF-β and promote M2 macrophage phenotypes, contributing to breast cancer progression. These exosomes also contain PD-L1 and semaphorins, which support the development of an immunosuppressive microenvironment [45]. Immunotherapy limits the production and functional activity of these exosomes, changing the way cancer cells communicate with their environment and supporting the immune response [45].

One of the key pleiotropic effects of checkpoint blockade relevant to VKH is the enhanced and sustained T cell activation. While this is beneficial for antitumor immunity, it can also break immune tolerance to melanocyte antigens. This heightened immune reactivity may lead to the formation of autoreactive memory T cells that persist after treatment, thereby contributing to the development of chronic autoimmune manifestations characteristic of VKH [42,43,44,45].

VKH-like uveitis has been reported in the literature in the context of ICIs and inhibitors of BRAF kinase and MEK kinase (BRAF/MEK inhibitors) [11]. Cases of VKH-like uveitis in women have been described more frequently in the literature, as is the case with idiopathic VKH [1]. In the case reported by Denu et al. [1], pembrolizumab was considered the most likely factor in the induction of VKH. A comprehensive summary of selected reported immune checkpoint inhibitor–induced VKH-like uveitis and panuveitis cases, including clinical presentation, management and outcomes, is provided in Table 2.

A retrospective analysis of cases of patients with VKH-like uveitis revealed that most common cancer treated was melanoma (69.2%), and standard treatment included the use of PD-1 inhibitors in monotherapy (63.5%). In patients diagnosed with melanoma, melanocytes have been observed to overexpress the PD-1 ligand. This is a process that prevents an immune response. The utilisation of PD-1 inhibitors has been demonstrated to abrogate this mechanism, consequently inducing a cross-reaction with melanocytes in the choroid of the eye. Consequently, this leads to an excessive immune response and damage [11]. An excessive immune response and loss of immune tolerance can also involve other organs containing melanocytes, such as the skin, the hearing organs, and the central nervous system [11].

Approximately 22 weeks after the initiation of immunotherapy for malignancy, patients developed symptoms characteristic of VKH, consistent with a treatment-related, drug-induced manifestation of the syndrome [11]. It is estimated that approximately 90% of patients required steroid treatment, and in more than half of them, immunotherapy had to be discontinued. Complete remission was achieved in approximately 83% of patients. As confirmed by analyses of clinical cases available in the literature, the antitumor response in patients with ICI-induced VKH was better [1]. According to Denu et al. [1], 8 out of 12 patients with melanoma achieved a complete or almost complete response to treatment, 2 patients had a partial response, and 2 patients achieved stabilisation of their health [1].

VKH syndrome is a condition that may occur as a side effect of immunotherapy. Untreated uveitis can result in permanent vision loss. Therefore, awareness of the risk of VKH-like uveitis in patients treated with ICIs is crucial [12].

## 6. Conclusions

VKH is an autoimmune disease that attacks melanocytes, and its classic clinical picture includes chronic uveitis and changes in the skin, inner ear, and meninges. Genetic predisposition, including polymorphisms of *HLA* genes and the IL-23/IL-17 pathway, as well as epigenetic factors, significantly increase the risk of developing both the classic and drug-induced forms of VKH. The administration of PD-1 inhibitor therapy has been observed to result in the onset of VKH-like uveitis in patients who are genetically predisposed to such conditions, underscoring the significance of the timely identification of risk factors. Understanding the immunogenetic determinants of the disease may help to improve diagnosis, risk prediction, and individualisation of care for patients treated with ICIs.

## Figures and Tables

**Table 1:** Stages of VKH syndrome and their clinical features [4,11,13,14].

VKH Stage	Main Clinical Symptoms
PRODROMAL	Lasts from several hours to several weeks Symptoms are mild Characterised by neurological and systemic flu-like symptoms Neurological deficits in the form of aphasia are present Symptoms of aseptic meningitis are present Dizziness, photophobia, and fever is present Meningeal symptoms such as neck stiffness and focal neurological symptoms are present Pleocytosis is found in the cerebrospinal fluid, which normalizes after administration of glucocorticosteroids
ACUTE CHOROIDITIS	This phase is characterised by a rapid deterioration in visual acuity, thickening of the choroid, or retinal detachment It can lead to serious retinal detachment and optic disc inflammation In addition, vitreous inflammation, optic disc edema, and the presence of Dalen-Fuchs nodules often co-occur Secondary to inflammatory infiltration of the ciliary body, there is a shift of the iridociliary diaphragm and narrowing of the filtration angle, which causes secondary glaucoma
RECURRENT	Appears after several weeks Characterised by depigmentation changes—periorbital albinism (Sugiyura’s sign), skin albinism, and choroidal changes known as “sunset glow fundus”—there is progressive depigmentation of the fundus and blanching of the optic disc, leading to orange-red discoloration Dalen-Fuchs nodules are typical and may be present in both the posterior pole and the periphery of the retina
CHRONIC	In the chronic phase, recurrent episodes of anterior segment inflammation, either granulomatous or non-granulomatous, are typical Complications such as glaucoma (33%), cataracts (50%), and choroidal neovascularization (10%) may develop Scarring and choroidal-retinal atrophy are present Sunset glow fundus changes persist Auditory symptoms—tinnitus, hearing disorders, hearing loss

**Table 2:** Reported immune checkpoint inhibitor–induced VKH-like uveitis and panuveitis cases and outcomes.

First Author	Year	Underlying Cancer	Drug	Exposure and Timing to Uveitis	Main Manifestations	Management of VKH-like Uveitis	Visual/Uveitis Outcome	Citations
Crosson JN	2015	metastatic melanoma	Ipilimumab (CTLA-4)	exact cycle timing not specified	retinal/choroidal pigment abnormalities, uveitis, headaches, auditory changes, diffuse cutaneous vitiligo, poliosis;	Observation routine uveitis surveillance; corticosteroids deferred	The patient remained under observation without systemic corticosteroid therapy, with no documented subsequent vision loss or recurrent intraocular inflammation	[46]
Tamura T	2018	non-small cell lung cancer (NSCLC)	Pembrolizumab (PD-L1)	PD-1 inhibitor 200 mg; after 3 cycles patient developed ocular pain and auditory changes	uveitis with ciliary hyperemia, granular leakage of fluorescein, optic disc leakage, OCT changes; sensorineural hearing loss; aseptic meningitis based on cerebrospinal fluid analysis	Systemic corticosteroid therapy; pembrolizumab discontinued	Uveitis improved after corticosteroid therapy	[12]
Wang JN	2024	bladder urothelial carcinoma	Toripalimab (PD-L1)	~20 months; onset 10 days after last dose	VKH-like panuveitis with exudative retinal detachment	Toripalimab permanently stopped; local (dexamethason sustained release implant) + systemic corticosteroids (prednisone)	Best-corrected visual acuity was 20/25 in both eyes, with no signs of active inflammation (January 2023)	[47]
Suwa S	2021	NSCLC	Atezolizumab (PD-L1)	~17 months	Severe bilateral uveitis	Atezolizumab discontinued; systemic steroids	Resolution within 2 months	[48]
Obata S	2019	metastatic cutaneous malignant melanoma	Nivolumab (PD-1)	after 10 days after the second injection	Bilateral panuveitis, serous retinal detachment, choroidal hyperfluorescence	Nivolumab discontinued; topical steroids, mydriatics	Best-corrected visual acuity recovered to 1.0 in the right eye and to 0.9 in the left eye; serous retinal detachment resolved by 3–4 months	[49]
Kikuchi R	2020	recurrent hypopharyngeal cancer	Nivolumab (PD-1)	after 2 cycles at a dose of 160 mg	Bilateral panuveitis, optic disc edema, diffuse serous retinal detachment	Sub-Tenon triamcinolone; intravenous steroid pulse then oral taper; nivolumab held	Retinal detachment resolved; rapid visual acuity improvement; no relapse at 3 months (patient later died of cancer)	[50]
Nagai R	2023	Gastric cancer	Nivolumab (PD-1)	~4 months after 8 cycles	Bilateral VKH-like uveitis with serous retinal detachment, wavy retinal pigment epithelium, cerebrospinal fluid pleocytosis, hearing loss	Topical + sub-Tenon triamcinolone; later oral prednisolone for hearing loss; no intravenous methylprednisolone	Serous retinal detachment/wavy retinal pigment epithelium changes resolved; stable at 1 year without relapse	[51]
Denu R	2024	Metastatic gastric cancer	Pembrolizumab (PD-1)	~18 months (maintenance)	Panuveitis, serous retinal detachment, bilateral uveal edema, secondary angle closure	Pembrolizumab stopped; local + systemic steroids	Visual acuity returned to baseline; uveitis resolved	[1]
Kontou E	2025	Metastatic colorectal cancer	Pembrolizumab (PD-1)	~5 months	Anterior uveitis, bilateral serous retinal detachment, optic disc edema, choroidal thickening	Oral steroids; pembrolizumab discontinued	Gradual clinical and visual acuity improvement	[52]
Nidha S	2025	Cutaneous squamous cell carcinoma (cSCC)	Cemiplimab (PD-1)	~3 months; after 4 cycles	Bilateral panuveitis, exudative retinal detachment, subretinal fibrosis	High-dose intravenous + topical steroids; cemiplimab stopped; oral taper	Significant anatomic and symptomatic improvement	[53]

## Data Availability

No new data were created or analyzed in this study.
